# The Effectiveness Of Social Media (Facebook) Compared With More Traditional Advertising Methods for Recruiting Eligible Participants To Health Research Studies: A Randomized, Controlled Clinical Trial

**DOI:** 10.2196/resprot.5747

**Published:** 2016-08-10

**Authors:** Mai Frandsen, Megan Thow, Stuart G Ferguson

**Affiliations:** ^1^ School of Health Sciences Faculty of Health University of Tasmania Launceston Australia; ^2^ School of Medicine Faculty of Health University of Tasmania Hobart Australia

**Keywords:** Facebook, recruitment methods, smoking, clinical trial

## Abstract

**Background:**

Recruiting participants for research studies can be difficult and costly. The popularity of social media platforms (eg, Facebook) has seen corresponding growth in the number of researchers turning to social networking sites and their embedded advertising frameworks to locate eligible participants for studies. Compared with traditional recruitment strategies such as print media, social media advertising has been shown to be favorable in terms of its reach (especially with hard-to-reach populations), cost effectiveness, and usability. However, to date, no studies have examined how participants recruited via social media progress through a study compared with those recruited using more traditional recruitment strategies.

**Objectives:**

(1) Examine whether visiting the study website prior to being contacted by researchers creates self-screened participants who are more likely to progress through all study phases (eligible, enrolled, completed); (2) compare conversion percentages and cost effectiveness of each recruitment method at each study phase; and, (3) compare demographic and smoking characteristics of participants recruited through each strategy to determine if they attract similar samples.

**Methods:**

Participants recruited to a smoking cessation clinical trial were grouped by how they had become aware of the study: via social media (Facebook) or traditional media (eg, newspaper, flyers, radio, word of mouth). Groups were compared based on throughput data (conversion percentages and cost) as well as demographic and smoking characteristics.

**Results:**

Visiting the study website did not result in individuals who were more likely to be eligible for (*P*=.24), enroll in (*P*=.20), or complete (*P*=.25) the study. While using social media was more cost effective than traditional methods when we examined earlier endpoints of the recruitment process (cost to obtain a screened respondent: AUD $22.73 vs $29.35; cost to obtain an eligible respondent: $37.56 vs $44.77), it was less cost effective in later endpoints (cost per enrolled participant: $56.34 vs $52.33; cost per completed participant: $103.66 vs $80.43). Participants recruited via social media were more likely to be younger (*P*=.001) and less confident in their quit attempts (*P*=.004) compared to those recruited via traditional methods.

**Conclusions:**

Our study suggests that while social media advertising may be effective in generating interest from potential participants, this strategy’s ability to attract conscientious recruits is more questionable. Researchers considering using online resources (eg, social media advertising, matrix codes) should consider including prescreening questions to promote conversion percentages. Ultimately, researchers seeking to maximize their recruitment budget should consider using a combination of advertising strategies.

**Trial Registration:**

Australian New Zealand Clinical Trials Registry ACTRN 12614000329662; https://www.anzctr.org.au/Trial/Registration/TrialReview.aspx?id=365947l (Archived by WebCite at http://www.webcitation.org/6jc6zXWZI)

## Introduction

### Background

One of the greatest challenges for researchers is recruiting eligible and representative participants to their studies. Traditionally, commonly used recruitment strategies include print media, radio, and informal channels such as word of mouth. More recently, researchers have turned to social media as an additional, or in many cases primary, recruitment strategy [1-3]. Compared with traditional recruitment strategies, social media appears attractive for its potential reach, apparent cost effectiveness, usability, and capacity for targeting hard to reach, isolated, and/or minority populations (eg, people with HIV [4,5], LGBT (lesbian, gay, bisexual, and transgender) populations [6], young women [7-10]). Ultimately, however, the utility of a recruitment strategy is defined by its ability to effectively attract people representative of the target population who are not only willing to participate in the study as per protocol but also to see the study to completion. Despite its popularity, data on social media recruitment’s effectiveness compared with more traditional methods is limited [11].

### Perceived Advantages of Social Media Advertising

Social media sites are vessels of vast amounts of personal data (based on user profiles) and are therefore extremely attractive to researchers, who can specifically target the audience of their advertisements (eg, users younger than 18 years living within a certain distance of a certain city). Indeed, Facebook and other social media platforms offer specifically generated and embedded advertising frameworks providing researchers more advanced, user-friendly, and data-generating recruitment platforms.

The enormous reach of social media platforms is another advantage of this recruitment strategy, with users all over the world representing a range of demographics. Facebook, for example, is one of the most visited sites on the Internet and the most popular social media site, with more than 1.5 billion monthly active users worldwide [12]. In years past the average Facebook user was a young woman under the age of 30, but there is evidence that the disparity in age and gender among Facebook users is lessening [13].

Another advantage of social media advertising platforms for recruitment is that researchers can exercise greater control over advertising duration and day-to-day expenditure compared to traditional recruitment strategies. Researchers can typically generate an ad, elect where (eg, mobile newsfeed, right-hand ad banners) and to whom it will be shown, predetermine how much they are willing to pay each time someone clicks on the ad, and indicate the overall daily budget they wish to spend on this advertising. Further, researchers can manipulate (eg, turn the ad on and off, increase the amount they are willing to pay per click) how many individuals are exposed to the advertising in near real time, controlling the flow of potential participants. In part because of this flexibility, social media has been suggested by a number of studies to be a cost-effective recruitment strategy [3,14,15] even if more expensive than other more traditional forms of advertising [16-18].

Finally, since people who click on an advertisement are redirected to a study website which may include more specific information about the study, eligibility criteria, full information sheet and/or further screening questions, individuals recruited via social media may be better informed. Our group [18] hypothesized that, by presenting detailed study information to interested individuals before they elect to participate, we attract individuals who are more likely to be eligible when contacted by researchers for screening, resulting in a higher conversion percentage both in terms of study completion and cost per participant. Testing these conversions is one of the main aims of this study.

### Potential Disadvantages of Social Media Advertising

In spite of the apparent potential benefits, social media advertising can only be considered a viable recruitment method if it is able to recruit representative samples of the target populations. For example, while social media advertising has been demonstrated to be extremely effective at recruiting participants to health studies specifically targeting young adults [9,14,19], other studies seeking to recruit a broader demographic sample (eg, for smoking cessation trials [18] or obtaining normative data for questionnaire development [20]) reported obtaining samples skewed to the younger demographic when using social media recruitment strategies. This reflects the fact that the average social media user is still at the younger end of the demographic spectrum and as such, may question social media’s ability to recruit more general population samples for health research and its overall effectiveness compared with more traditional recruitment strategies.

To date, however, few studies have compared the demographic characteristics of samples collected via social media to those recruited via traditional strategies. In a previous study [18], our group reported that participants recruited via social media were significantly younger than those recruited via traditional media and thus cautioned against solely relying on social media advertising to recruit for studies targeting more diverse population samples. As we noted in the original study, however, this finding needs to be replicated.

Finally, we have previously reported that the cost of participants recruited via social media was almost twice as expensive as those recruited via traditional recruitment strategies; however, we cautioned that this was not a true indication of cost-effectiveness because other contributing factors could not be accounted for in this calculation (eg, cost of personnel screening participants, conversion percentages of enrolled and completed participants [18]).

### This Study

In light of the popularity and reporting of social media advertising for health research, this study aims to replicate our 2014 research [18] by considering whether social media is more cost-effective than traditional strategies in recruiting for health research and whether samples recruited via social media reflect those recruited via traditional avenues. Since the success of a study is not determined by how many participants are recruited but by how many actually comply with study protocol and complete the study, we compare the conversion percentages of participants at each phase of the study (screened, eligible, enrolled, and completed) with how they were recruited. This way, we offer a more detailed interpretation of the effectiveness of social media (Facebook) as a recruitment strategy to health research compared with traditional media (newspaper, radio, flyers, word of mouth). Specifically, we aim to:

Examine whether individuals who visit the study website are more likely, due to self-screening, to be eligible for, enroll in, and complete the study.

Compare conversion percentages and cost effectiveness of individuals recruited through social media with those recruited through traditional media at each study phase (ie, screened, eligible, enrolled, and completed) to determine if either method is more efficacious.

Compare demographic and smoking characteristic data of participants recruited through social media with traditional media to determine if representativeness of the target sample is comparable between recruitment strategies.

## Methods

### Overview

Data for this study are a subset drawn from a larger study investigating the mechanism through which smoking cessation medications promote abstinence [21]. Results of this randomized, open-label controlled clinical trial will be reported elsewhere. Here we describe the recruitment process and present data on the effectiveness of social media advertising compared with traditional media advertising methods in recruiting a representative sample of interested quitters to a smoking cessation trial.

### Target Participants

Participants were adult smokers who reported smoking 10 or more cigarettes per day (CPD) for the past 3 years and indicated no intention to quit within the next month. Participants were excluded if they reported current or recent (within the last 3 months) participation in a smoking cessation program or had existing medical conditions (eg, epilepsy, diabetes, depression) that deemed them unsuitable for treatment using nicotine patches or varenicline. Participants were recruited between September 23, 2014, and November 9, 2015. The study was approved by the Tasmanian Health and Medical Human Research Ethics Committee (H0013619).

### Recruitment Strategies

A combination of traditional media advertising strategies including newspaper ads, flyers, radio, word of mouth, and paid social media advertising on Facebook were used concurrently to recruit participants to this study. Both newspaper and flyer ads contained brief information about the study including contact details of researchers and a matrix barcode which could be scanned to direct interested people to the study website if they wanted more information. Flyers were distributed at University of Tasmania campuses and surrounding shopping districts.

Multiple Facebook ads were created using a combination of wording and images and were rotated and switched on and off in response to recruitment flow. Facebook ads were set up to target adults (18 years and older) living within 25 kilometers of the recruitment site. Interested individuals who clicked on the Facebook advertisement were automatically redirected to a study website containing a brief description of the study and a link to the study information sheet. Interested individuals were then prompted to enter their contact details, which were automatically forwarded to the study researchers who subsequently contacted these individuals for participation screening. We set social media daily spending targets—typically capped at AUD $30 per day—with advertisements regularly turned on and off over the course of the week in an attempt to reduce advertisement fatigue.

### Procedure

The process of recruitment occurred in four phases. Individuals screened were all those who registered interest in the study (eg, via phone, internet, word of mouth) and who were subsequently contacted by the researchers via a telephone call to confirm eligibility. During the screening process, data were collected including how they had heard of the study, whether they had seen the ad themselves or someone had told them about it, and whether they had visited the study website prior to contacting/being contacted by the research team. Screening criteria were then assessed (eg, intention to quit, conflicting medical condition, agreement to use treatment) and person’s eligibility determined. Interested individuals were considered *eligible* for the study if they met the eligibility criteria. Individuals were *enrolled* once they had completed an enrollment session and were considered a participant of the study. At enrollment, participants provided baseline demographic and smoking characteristic data before completing subsequent study procedures. Participants had *completed* the study once they had finished the full study protocol, which included study visits over 6 weeks, 4 to 6 weeks of either nicotine patch or varenicline treatment to assist with quitting smoking, and 2 weeks of ecological momentary assessment to track affect/craving/behavior (full details of the trial are reported elsewhere [21]). Thus, data detailing participants who completed the study are presented as retention percentages in the present paper.

**Table 1:** Screened individuals by recruitment strategy (N=414).

Recruitment strategy		n (%)
**Social media**	Facebook	228 (55.1)
**Traditional media**		148 (37.5)
	Newspaper	92 (22.2)
	Word of mouth	33 (8.0)
	Flyer	22 (5.3)
	Internet^a^	4 (1.0)
	Radio	1 (0.2)
	Unknown^a^	34 (8.2)

^a^Internet and Unknown were excluded from further comparisons.

### Data Reduction and Analytical Plan

In total, 414 interested individuals were screened for study eligibility. To explore differences between individuals reached by different recruitment methods, screened individuals were grouped by how they had heard of the study (Table 1): Facebook, Internet, newspaper, radio, flyer, and word of mouth.

A total of 34 individuals did not indicate how they had heard of the study, allowing 380 to be categorized by recruitment method. Individuals who reported hearing about the study via flyer, radio, word of mouth, and newspaper were categorized as being reached via traditional media; individuals who heard about the study via Facebook were categorized as being reached via social media. Four individuals indicated becoming aware of the study via the Internet. Because it could not be determined whether this was indeed Facebook or another website, these data were not included in the traditional media versus social media comparisons, thus resulting in a final sample of 376 (with 38 of the original 414 excluded).

To examine the first aim of the study, all individuals screened for eligibility who had visited the website prior to contacting researchers were compared at each phase of the study (eligible, enrolled, and completed) using a series of chi-square tests. Similarly, the number of and cost per participant at each study phase were compared in order to explore conversion percentages and cost effectiveness of social media versus traditional media (Aim 2). Finally, to determine whether participants recruited via social media generally reflected those recruited via traditional media (Aim 3), the demographic (age, gender, income, and education) and smoking characteristics (CPD, motivation and confidence to quit, Heaviness of Smoking Index [HSI], and number of past quit attempts) of each group were compared using independent samples *t* tests and chi-squares.

## Results

### Objective 1: Comparison of Individuals Visiting the Study Website by Proportion Who Are Eligible for, Enroll in, and Complete the Study

Table 2 compares the proportions of individuals who visited the study website at each phase of the study to those who did not. Those who visited the website prior to contacting the research team were not more likely to be eligible (*P*=.24), be successfully enrolled (*P*=.20), or complete the study *(P*=.25). In addition, we examined the proportion of individuals who visited the study website within each recruitment method. Not surprisingly, fewer participants recruited through traditional media (52/148, 35.5%) reported visiting the study website compared to social media–recruited participants (228, 100%) (*P*<.001).

**Table 2:** Proportion of recruited interested individuals (n=353) who visited the study website prior to screening.

	Visited website prior to contacting researchers	
	Yes n (%)	No n (%)
Overall (screened)^a^	284 (80.5)	69 (19.5)
Eligible^b^	176 (62.0)	48 (69.6)
Enrolled^b^	128 (45.1)	37 (53.6)
Completed^b^	75 (26.4)	23 (33.3)

^a^n=61 did not report whether they had visited website.

^b^Proportion of overall who had and had not visited the website.

### Objective 2: Comparison of Conversion Percentages and Cost Effectiveness of Each Recruitment Method

Table 3 provides a breakdown of proportions of individuals interested in the study by recruitment phase (screened, eligible, enrolled, and completed) and media strategy as well as cost per participant at each of these study phases. Costs associated with Facebook advertising were drawn from the study’s Facebook advertising manager matrices and cross-checked with monthly credit card expenditure as charged and invoiced to the study’s account. Total cost of Facebook advertising over the course of the study was $5183.13. Traditional media costs comprise 12 individual ads to a local newspaper at $313.80 plus production costs ($288.75) for the two ads, totaling $4343.10. Cost associated with the printing and distribution of flyers was not recorded, and radio interviews were conducted free of charge. As such, the total cost of traditional media advertising reflects only the cost associated with newspaper advertising. Compared with individuals recruited through social media, a greater proportion of those who became aware of the study via traditional media were eligible, enrolled into, and completed the study (Table 3). Furthermore, while social media advertising captured more initial recruits at a lower cost, the cost per participant was less at the enrolled and completed stages for traditional media recruited participants.

**Table 3:** Cost of participant by recruitment strategy and study phase.

	Social media		Traditional media	
Total cost	AUD $5183.13		AUD $4343.10	
	n (%)	$	n (%)	$
Screened	228 (100)	22.73	148 (100)	29.35
Eligible	138 (60.5)	37.56	97 (65.5)	44.77
Enrolled	92 (40.4)	56.34	83 (56.1)	52.33
Completed	50 (21.9)	103.66	54 (36.5)	80.43

### Objective 3: Comparison of Demographic Profile of Enrolled Participants

Significant differences between recruitment groups were found for age and quitting characteristics (Table 4). Participants recruited via social media were more likely to be younger and self-report as less confident in their ability to quit in comparison with those recruited through traditionally media. There were no differences in gender, education, income, CPD, motivation, HSI, or number of past quit attempts.

**Table 4:** Demographic and smoking characteristics of enrolled participants: traditional media versus social media.

			Overall n=182	Social media n=92	Traditional media n=83	*P* value
**Demographics**						
	Age, years, mean (SD^a^)		42.3 (12.0)	39.3 (10.9)	44.9 (12.6)	.01
	Gender (female)^b^, n (%)		71 (40.8)	43 (46.7)	28 (34.1)	.09
	**Education^c^, n (%)**						
		High school or less, n (%)	74 (45.4)	39 (45.9)	35 (44.9)	.90
		Certificate or trade, n (%)	60 (36.)	34 (40.0)	26 (33.3)	.38
		College, n (%)	29 (17.8)	12 (14.1)	17 (21.8)	.20
	**Income^d^, n**					
		<$21,000	18 (10.9)	6(7.1)	12(15.0)	.10
		$21,000-$51,999	38 (23.0)	19 (22.4)	19 (23.8)	.83
		$52,000-$77,999	38 (23.0)	24 (28.2)	14 (17.5)	.10
		$78,000-$103,999	31 (18.8)	16 (18.8)	15 (18.8)	.99
		>$104,000	40 (24.2)	20 (23.5%)	20 (25.0)	.83
**Smoking characteristics**						
	CPD, mean (SD)		18.2 (7.0)	19.1 (8.0)	17.4 (5.8)	.14
	Motivation to quit, mean (SD)		89.5 (10.8)	89.2 (8.0)	91.1 (8.8)	.23
	Confidence to quit, mean (SD)		72.6 (19.3)	69.2 (19.7)	77.3 (16.4)	<.01
	HSI, mean (SD)		3.0 (1.2)	3.2 (1.3)	2.9 (1.2)	.19
	Number of past quit attempts, mean (SD)		3.8 (3.9)	4.1 (4.4)	3.3 (2.8)	.14

^a^SD: standard deviation.

^b^n=174 (8 missing).

^c^n=163 (19 missing).

^d^n=165 (17 missing).

## Discussion

### Principal Findings

The main findings of this study were that visiting the study website did not result in individuals who were more likely to be eligible for, enroll in, or complete the study. While using social media drew more interest and was more cost effective than traditional methods when we examined earlier endpoints of the recruitment process (ie, screened and eligible), it was less cost effective in later endpoints (enrolled and completed). Participants recruited via social media were more likely to be younger and less confident in their quit attempts compared to those recruited via traditional methods. There were no other demographic or smoking characteristic differences between individuals by recruitment strategy.

Although the popularity of social media recruitment for health research is increasing, data (including cost effectiveness and conversion and retention percentages) on this strategy’s effectiveness and efficiency compared with more traditional recruitment are limited [11]. Here we address this by reporting on the conversion and retention percentages of participants in a smoking cessation study. Specifically, we examined whether participants recruited via social media are more likely to be eligible, enroll in, and complete the study due to self-screening. The apparent cost effectiveness of social media over traditional media advertising was also examined. Finally, the study compared the demographic characteristics of participants recruited via social media and traditional media.

The first finding of the study was that interested individuals who visited the study website, regardless of the recruitment method, prior to contacting the research team were not more likely to be eligible for participation in our study. As such, using social media advertising like Facebook or including tools such as matrix codes on more traditional recruitment mediums as we did in our study did not appear to promote participant self-screening or study conversion. One explanation for this may be that while interested individuals are automatically directed or self-direct (using the matrix code) to study information via a website, they do not necessarily read the materials and thus effectively self-screen. Researchers contemplating using a study website to boost self-screening should consider incorporating prescreening questions requiring interested people to answer a series of questions correctly (based on the embedded study information) before being allowed to enter their contact details.

Interestingly, while all participants recruited via Facebook in this study were coded as having visited the website prior to contacting researchers (by clicking on the Facebook ads, these individuals were automatically directed to the study website), when asked by the researcher during the screening telephone call whether they had indeed visited the website, only approximately two-thirds (146/228, 64.0%) indicated that they had. This suggests that these participants were not aware of having been directed to the website and had not read the study information. Future studies should explore methods of improving the use of online resources such as prescreening questions prior to allowing interested individuals to enter their contact details to ensure those not eligible are identified as early as possible.

The second finding of our research was that while social media advertising captured more individuals interested in the study, they were not more likely to be eligible, enrolled into, or complete the study compared with those recruited through traditional media. This was surprising given these individuals were automatically directed to the study website where it was assumed they would read some information before providing details to researchers for follow-up screening and enrolment. This result implies that the use of social media does not lead to better informed and potentially self-screened participants compared with traditional media. In terms of cost effectiveness, while social media provided recruits at a lower cost at the screening and eligibility phases of the study, the cost per participant was more at the enrolled and completed phases of the study. Overall, not only did traditional media capture a greater proportion of participants who were eligible, enrolled into, and completed the study, this medium was also more cost effective in the latter two phases of the study. Similar findings were reported by Rait et al [17] who also compared cost and conversion rates of participants recruited to a smoking cessation study. They found that although Facebook attracted higher numbers of interested individuals to the study, Facebook recruitment had a higher ineligibility rate and was less cost effective to enroll participants than using traditional media.

It is possible that social media attracts individuals who click on an ad in the spur of the moment. Facebook advertisements reach their audiences by popping up in user newsfeeds and thus, if interested, the individual has to click on the advertisement there and then or it disappears. As such, social media advertising may attract people who have not otherwise given quitting that much thought but on the spur of the moment decide to enter their details allowing the researchers to contact them (as is the case for traditionally recruited participants who scan the matric code, are directed to website, and if interested, enter their contact details). This is in part supported by our finding that participants recruited through social media advertising were less confident in their ability to quit compared to those recruited through traditional media. Further, interested people who see a newspaper advertisement, hear a radio advertisement, or see a flyer may be more likely to contact the researchers themselves (eg, leave a message on answering machine) and may have put more thought and deliberation into their decision to participate, potentially resulting in a more conscientious participant.

It has also been suggested that social media users are more likely to suffer from mental health conditions [22], and we might expect that respondents via this medium would be more likely to be deemed ineligible for the study (as per study protocol, participants with existing mental health conditions were not eligible to participate), providing some explanation to the differing conversion percentages. However, no differences were found between the participants recruited by social media (44/228, 19.3%) and traditional media (27/148, 18.2%) who indicated having an existing mental health condition.

The third finding of this research was that the demographic profile and smoking characteristics of participants recruited via social media largely mirrored that of those recruited through traditional media. While individuals recruited and enrolled through social media were more likely to be younger and less confident in their quit attempt, no differences were found in other demographic or smoking characteristics. This in part supports the findings of other studies [18,20] which have shown that participants recruited via social media were more likely to be young and female. That this study did not find a significant difference in the proportion of males to females recruited by recruitment strategy supports the trend of social media user profiles increasingly representing a broader demographic. This finding therefore provides optimism for the utility of social media sites like Facebook to recruit more representative samples in the future.

### Limitations

While informative, this study is not without limitations. First, while the direct cost of newspaper advertising is known, the actual cost of participants recruited through other traditional means like flyers is unknown. For example, the time spent and associated researcher costs of distributing flyers and screening interested individuals via telephone is unknown. Similarly, researcher time spent (and thus cost) of monitoring and managing Facebook advertising was not recorded but is likely comparable to that of managing recruitment flow of more traditional recruitment strategies. In addition, it is noted that 8% (34/414) of participants did not indicate how they became aware of the study and were not able to be categorized into either social media or traditional media. However, the proportion is relatively small and hence unlikely to influence the results.

This study limited its social media advertising to Facebook, and while this was a deliberate choice because Facebook is the most popular and far-reaching social media site on the Internet, our results may not be as readily generalized to other social media platforms. It is possible although unlikely that other social media platform users may provide more conscientious participants. Furthermore, we only used paid Facebook advertising. Other studies have reported on the efficacy of social media and specifically Facebook as a free advertising and recruitment tool (eg, placing ads on certain social pages or creating a free Facebook page for the study) [23,24]. Researchers considering using social media for health research may like to consider both paid and unpaid methods of advertising to fully exploit the benefits these frameworks offer.

### Future Research

As Lane and colleagues [11] state, this study stands alone in its attempts to report on the effectiveness and efficiency, using empirical data as evidence, of social media advertising compared with more traditional means in recruiting participants who will complete the health studies to which we recruit them. As such, the findings we have presented here should be interpreted with regard to the target sample sought (Australian smokers wishing to quit) and advertising methods used. Until other studies provide empirical data (retention and cost conversation rates/percentages) on the effectiveness and efficiency of social media recruitment compared with traditional media recruitment, we encourage health researchers wishing to maximize recruitment to their studies to use a combination of social media and traditional recruitment advertising strategies. However, we also caution that while social media platforms such as Facebook may be effective in recruiting large numbers of participants to a study, these individuals may represent less conscientious participants, resulting in lower conversion rates and more expensive participants compared to traditional media platforms. Future studies should consider including embedded prescreening questions to check if people directed to study websites actually read the information they are assumed to. Future research should also explore the earlier suggestion and implications that participants recruited through social media may have higher rates of mental health issues.

### Conclusions

Social media advertising is an effective and user-friendly recruitment strategy for reaching a large sample at a comparatively lower cost than traditional media recruitment strategies. However, researchers must be aware that samples recruited solely through social media may be demographically skewed. In the long term, social media–recruited participants may not be as representative of the target population or as conscientious as participants recruited via traditional media. To our knowledge, this study is the first to report on the efficacy of social media advertising compared with more traditional media recruitment strategies to attract demographic samples who will cost effectively and successfully complete study participation. Until further studies are reported, we would advise researchers to use a combination of recruitment strategies to maximize reach, retention, and target-sample representativeness.

## Abbreviations

CPD: cigarettes per day
HSI: Heaviness of Smoking Index
SD: standard deviation
